# 4-Dimethyl­amino-*N*′-(2-hy­droxy-4-meth­oxy­benzyl­idene)benzohydrazide methanol monosolvate

**DOI:** 10.1107/S1600536812039906

**Published:** 2012-09-26

**Authors:** Jingya Sun

**Affiliations:** a College of Marine Sciences, Zhejiang Ocean University, Zhoushan 316000, People’s Republic of China

## Abstract

The asymmetric unit of the title compound, C_17_H_19_N_3_O_3_·CH_4_O, comprises two Schiff base mol­ecules and two methanol solvent mol­ecules. The Schiff base mol­ecules are approximately planar, with r.m.s. deviations from the planes defined by the non-H atoms of 0.107 and 0.154 Å, and with dihedral angles between the benzene rings of 4.49 (15) and 8.39 (15)°, respectively. This near-planarity is assisted by the formation of intra­molecular O—H⋯N hydrogen bonds in each mol­ecule. In the crystal, the components are linked by N—H⋯O and O—H⋯O hydrogen bonds to form chains along [010].

## Related literature

For the properties of Schiff base compounds, see: Miura *et al.* (2009 ▶); Zhao *et al.* (2010 ▶); Karadağ *et al.* (2011 ▶); Bingöl Alpaslan *et al.* (2010 ▶). For the structure of a related Schiff base compound, see: Xu & Sun (2012 ▶).
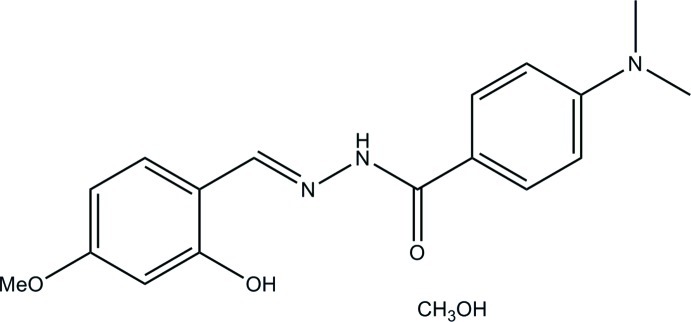


## Experimental

### 

#### Crystal data

C_17_H_19_N_3_O_3_·CH_4_O
*M* *_r_* = 345.39
Monoclinic, 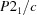

*a* = 7.7426 (17) Å
*b* = 23.473 (2) Å
*c* = 20.1975 (16) Å
β = 100.495 (2)°
*V* = 3609.3 (9) Å^3^
*Z* = 8
Mo *K*α radiation
μ = 0.09 mm^−1^
*T* = 298 K
0.17 × 0.15 × 0.15 mm

#### Data collection

Bruker SMART CCD diffractometer
Absorption correction: multi-scan (*SADABS*; Bruker, 2001 ▶) *T*_min_ = 0.985, *T*_max_ = 0.987
17259 measured reflections
6668 independent reflections
2824 reflections with *I* > 2σ(*I*)
*R*_int_ = 0.051

#### Refinement

*R*[*F* ^2^ > 2σ(*F* ^2^)] = 0.052
*wR*(*F* ^2^) = 0.155
*S* = 0.99
6668 reflections
471 parameters
3 restraints
H atoms treated by a mixture of independent and constrained refinement
Δρ_max_ = 0.15 e Å^−3^
Δρ_min_ = −0.16 e Å^−3^

### 

Data collection: *SMART* (Bruker, 2007 ▶); cell refinement: *SAINT* (Bruker, 2007 ▶); data reduction: *SAINT*; program(s) used to solve structure: *SHELXTL* (Sheldrick, 2008 ▶); program(s) used to refine structure: *SHELXTL*; molecular graphics: *SHELXTL*; software used to prepare material for publication: *SHELXTL*.

## Supplementary Material

Crystal structure: contains datablock(s) global, I. DOI: 10.1107/S1600536812039906/hb6963sup1.cif

Structure factors: contains datablock(s) I. DOI: 10.1107/S1600536812039906/hb6963Isup2.hkl

Supplementary material file. DOI: 10.1107/S1600536812039906/hb6963Isup3.cml

Additional supplementary materials:  crystallographic information; 3D view; checkCIF report

## Figures and Tables

**Table 1:** Hydrogen-bond geometry (Å, °)

*D*—H⋯*A*	*D*—H	H⋯*A*	*D*⋯*A*	*D*—H⋯*A*
O1—H1⋯N1	0.82	1.86	2.588 (3)	147
O4—H4⋯N4	0.82	1.93	2.649 (3)	146
O7—H7⋯O5	0.82	1.87	2.671 (3)	166
O8—H8⋯O2^i^	0.85 (1)	1.86 (1)	2.713 (3)	179 (4)
N5—H5⋯O8^ii^	0.91 (1)	2.02 (1)	2.892 (4)	161 (3)
N2—H2⋯O7^iii^	0.90 (1)	2.01 (2)	2.876 (3)	159 (3)

Symmetry codes: (i) 
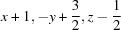
; (ii) 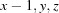; (iii) 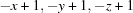.

**Fig. 1.**
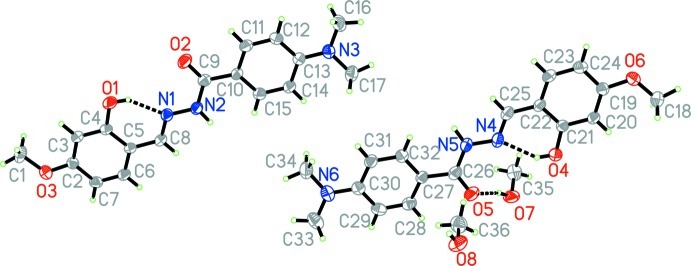
The molecular structure of the title compounds with 30% probability displacement ellipsoids. Hydrogen bonds are shown as dashed lines.

**Fig. 2.**
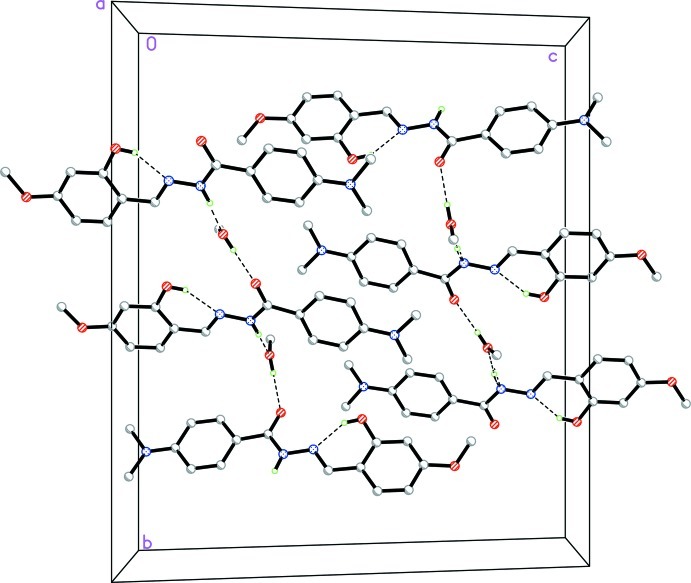
The molecular packing of the title compound. Hydrogen bonds are shown as dashed lines.

## supplementary crystallographic information

### Comment

The condensation reaction between aldehydes with organic primary amines readily forms Schiff bases containing the typical –C=N– groups (Miura *et al.*, 2009; Zhao *et al.*, 2010; Karadağ *et al.*, 2011; Bingöl Alpaslan *et al.*, 2010). As a continuation of our work on Schiff bases (Xu & Sun, 2012), in this paper, the title new compound (Fig. 1), prepared by the reaction of 4-methoxysalicylaldehyde with 4-dimethylaminobenzohydrazide in methanol, is reported.

The asymmetric unit of the compound comprises two Schiff base molecules, A and B, and two methanol molecules. The Schiff base molecules are approximately planar, with r.m.s. deviations from the planes defined by the non-hydrogen atoms of 0.107 and 0.154 Å, and with dihedral angles between the benzene rings of 4.49 (15) and 8.39 (15)° for molecules A and B, respectively. This planarity is assisted by the formation of intramolecular O—H···N hydrogen bonds (Table 1). In the crystal, (Fig. 2), Schiff base molecules are linked by methanol molecules through N—H···O and O—H···O hydrogen bonds (Table 1) to form chains.

### Experimental

4-Methoxysalicylaldehyde (1.0 mmol, 0.152 g) and 4-dimethylaminobenzohydrazide (1.0 mmol, 0.179 g) were refluxed for 30 min in 30 ml methanol, and cooled to room temperature to give colorless solid, which was isoloated by filtration. Colourless blocks were formed by recrystallization of the solid product in methanol.

### Refinement

H2, H5, and H8 were located from a difference Fourier map and refined isotropically, with N—H and O—H distances restained to 0.90 (1) and 0.85 (1) Å, respectively. The remaining hydrogen atoms were positioned geometrically and refined using the riding-model approximation, with C—H = 0.93–0.96 Å, and O—H = 0.82 Å, and *U*_iso_(H) = 1.2*U*_eq_(C) or *U*_iso_(H) = 1.5*U*_eq_(O and methyl C).

### Crystal data

**Table:**

C_17_H_19_N_3_O_3_·CH_4_O	*F*(000) = 1472
*M**_r_* = 345.39	*D*_x_ = 1.271 Mg m^−^^3^
Monoclinic, *P*2_1_/*c*	Mo *K*α radiation, λ = 0.71073 Å
*a* = 7.7426 (17) Å	Cell parameters from 2668 reflections
*b* = 23.473 (2) Å	θ = 2.2–24.5°
*c* = 20.1975 (16) Å	µ = 0.09 mm^−^^1^
β = 100.495 (2)°	*T* = 298 K
*V* = 3609.3 (9) Å^3^	Block, colorless
*Z* = 8	0.17 × 0.15 × 0.15 mm

### Data collection

**Table:**

Bruker SMART CCD diffractometer	6668 independent reflections
Radiation source: fine-focus sealed tube	2824 reflections with *I* > 2σ(*I*)
Graphite monochromator	*R*_int_ = 0.051
ω scans	θ_max_ = 25.5°, θ_min_ = 2.2°
Absorption correction: multi-scan (*SADABS*; Bruker, 2001)	*h* = −8→9
*T*_min_ = 0.985, *T*_max_ = 0.987	*k* = −27→28
17259 measured reflections	*l* = −24→18

### Refinement

**Table:**

Refinement on *F*^2^	Primary atom site location: structure-invariant direct methods
Least-squares matrix: full	Secondary atom site location: difference Fourier map
*R*[*F*^2^ > 2σ(*F*^2^)] = 0.052	Hydrogen site location: inferred from neighbouring sites
*wR*(*F*^2^) = 0.155	H atoms treated by a mixture of independent and constrained refinement
*S* = 0.99	*w* = 1/[σ^2^(*F*_o_^2^) + (0.0599*P*)^2^] where *P* = (*F*_o_^2^ + 2*F*_c_^2^)/3
6668 reflections	(Δ/σ)_max_ < 0.001
471 parameters	Δρ_max_ = 0.15 e Å^−^^3^
3 restraints	Δρ_min_ = −0.16 e Å^−^^3^

### Special details

**Table:**

Geometry. All e.s.d.'s (except the e.s.d. in the dihedral angle between two l.s. planes) are estimated using the full covariance matrix. The cell e.s.d.'s are taken into account individually in the estimation of e.s.d.'s in distances, angles and torsion angles; correlations between e.s.d.'s in cell parameters are only used when they are defined by crystal symmetry. An approximate (isotropic) treatment of cell e.s.d.'s is used for estimating e.s.d.'s involving l.s. planes.
Refinement. Refinement of *F*^2^ against ALL reflections. The weighted *R*-factor *wR* and goodness of fit *S* are based on *F*^2^, conventional *R*-factors *R* are based on *F*, with *F* set to zero for negative *F*^2^. The threshold expression of *F*^2^ > σ(*F*^2^) is used only for calculating *R*-factors(gt) *etc*. and is not relevant to the choice of reflections for refinement. *R*-factors based on *F*^2^ are statistically about twice as large as those based on *F*, and *R*- factors based on ALL data will be even larger.

### Fractional atomic coordinates and isotropic or equivalent isotropic displacement parameters (Å^2^)

**Table:**

	*x*	*y*	*z*	*U*_iso_*/*U*_eq_	
N1	0.3235 (3)	0.69983 (10)	0.90099 (12)	0.0533 (7)	
N2	0.2735 (3)	0.69032 (10)	0.83293 (13)	0.0554 (7)	
N3	−0.0477 (3)	0.68909 (11)	0.51462 (14)	0.0602 (7)	
N4	0.3574 (3)	0.54206 (11)	0.19498 (13)	0.0604 (7)	
N5	0.3984 (4)	0.55391 (11)	0.26325 (14)	0.0612 (7)	
N6	0.7055 (4)	0.57953 (12)	0.58081 (15)	0.0696 (8)	
O1	0.3173 (3)	0.75604 (9)	1.01077 (10)	0.0795 (7)	
H1	0.2915	0.7486	0.9705	0.119*	
O2	0.0887 (3)	0.76477 (9)	0.82402 (10)	0.0768 (7)	
O3	0.6142 (3)	0.67343 (9)	1.21576 (11)	0.0720 (7)	
O4	0.3881 (3)	0.49011 (10)	0.08124 (10)	0.0745 (7)	
H4	0.4112	0.4965	0.1218	0.112*	
O5	0.6060 (3)	0.48503 (10)	0.27995 (11)	0.0850 (8)	
O6	0.0622 (3)	0.56832 (9)	−0.12049 (11)	0.0744 (7)	
O7	0.4931 (3)	0.39331 (12)	0.20641 (14)	0.1030 (9)	
H7	0.5105	0.4225	0.2290	0.154*	
O8	1.1189 (4)	0.62247 (10)	0.29899 (13)	0.0873 (8)	
C1	0.5549 (6)	0.71737 (17)	1.25440 (18)	0.1079 (14)	
H1A	0.5902	0.7537	1.2395	0.162*	
H1B	0.6052	0.7122	1.3011	0.162*	
H1C	0.4291	0.7160	1.2488	0.162*	
C2	0.5658 (4)	0.67530 (13)	1.14705 (16)	0.0512 (8)	
C3	0.4607 (4)	0.71645 (13)	1.11238 (16)	0.0544 (8)	
H3	0.4181	0.7460	1.1355	0.065*	
C4	0.4182 (4)	0.71381 (12)	1.04274 (16)	0.0512 (8)	
C5	0.4783 (4)	0.66948 (12)	1.00711 (15)	0.0454 (7)	
C6	0.5853 (4)	0.62875 (12)	1.04426 (16)	0.0543 (8)	
H6	0.6275	0.5988	1.0217	0.065*	
C7	0.6304 (4)	0.63133 (13)	1.11282 (17)	0.0580 (9)	
H7A	0.7038	0.6039	1.1363	0.070*	
C8	0.4254 (4)	0.66316 (12)	0.93510 (15)	0.0506 (8)	
H8A	0.4656	0.6323	0.9134	0.061*	
C9	0.1532 (4)	0.72512 (13)	0.79644 (16)	0.0527 (8)	
C10	0.1034 (4)	0.71363 (12)	0.72368 (15)	0.0473 (7)	
C11	−0.0104 (4)	0.75043 (13)	0.68370 (16)	0.0564 (8)	
H11	−0.0556	0.7813	0.7038	0.068*	
C12	−0.0592 (4)	0.74306 (13)	0.61545 (16)	0.0568 (8)	
H12	−0.1351	0.7692	0.5907	0.068*	
C13	0.0023 (4)	0.69717 (12)	0.58217 (16)	0.0477 (8)	
C14	0.1173 (4)	0.65991 (13)	0.62233 (16)	0.0557 (8)	
H14	0.1623	0.6289	0.6024	0.067*	
C15	0.1656 (4)	0.66801 (13)	0.69059 (16)	0.0569 (8)	
H15	0.2423	0.6422	0.7155	0.068*	
C16	−0.1515 (5)	0.73097 (15)	0.47253 (16)	0.0873 (12)	
H16A	−0.2615	0.7361	0.4873	0.131*	
H16B	−0.1727	0.7181	0.4266	0.131*	
H16C	−0.0892	0.7665	0.4757	0.131*	
C17	0.0302 (4)	0.64438 (14)	0.48000 (16)	0.0737 (10)	
H17A	0.1555	0.6490	0.4880	0.111*	
H17B	−0.0147	0.6467	0.4325	0.111*	
H17C	0.0013	0.6079	0.4965	0.111*	
C18	0.1354 (5)	0.52835 (15)	−0.16021 (16)	0.0790 (11)	
H18A	0.2609	0.5322	−0.1518	0.118*	
H18B	0.0903	0.5353	−0.2070	0.118*	
H18C	0.1045	0.4905	−0.1487	0.118*	
C19	0.1098 (4)	0.56550 (13)	−0.05226 (16)	0.0534 (8)	
C20	0.2268 (4)	0.52696 (12)	−0.01860 (15)	0.0510 (8)	
H20	0.2775	0.4997	−0.0424	0.061*	
C21	0.2693 (4)	0.52866 (12)	0.05111 (15)	0.0489 (8)	
C22	0.1962 (4)	0.56981 (13)	0.08811 (16)	0.0508 (8)	
C23	0.0771 (4)	0.60764 (14)	0.05194 (18)	0.0653 (9)	
H23	0.0263	0.6352	0.0753	0.078*	
C24	0.0313 (4)	0.60594 (14)	−0.01701 (18)	0.0686 (10)	
H24	−0.0508	0.6314	−0.0397	0.082*	
C25	0.2455 (4)	0.57557 (14)	0.15955 (17)	0.0580 (9)	
H25	0.1950	0.6045	0.1811	0.070*	
C26	0.5289 (4)	0.52472 (15)	0.30257 (17)	0.0611 (9)	
C27	0.5723 (4)	0.54179 (13)	0.37413 (15)	0.0506 (8)	
C28	0.6851 (4)	0.50770 (13)	0.41801 (18)	0.0608 (9)	
H28	0.7325	0.4756	0.4011	0.073*	
C29	0.7299 (4)	0.51937 (14)	0.48550 (18)	0.0628 (9)	
H29	0.8064	0.4952	0.5131	0.075*	
C30	0.6623 (4)	0.56715 (13)	0.51346 (17)	0.0538 (8)	
C31	0.5490 (4)	0.60199 (13)	0.46922 (16)	0.0587 (9)	
H31	0.5019	0.6343	0.4859	0.070*	
C32	0.5057 (4)	0.58973 (13)	0.40179 (16)	0.0582 (8)	
H32	0.4301	0.6140	0.3738	0.070*	
C33	0.8215 (5)	0.54243 (16)	0.62615 (17)	0.0867 (12)	
H33A	0.7738	0.5046	0.6236	0.130*	
H33B	0.8325	0.5564	0.6714	0.130*	
H33C	0.9351	0.5418	0.6135	0.130*	
C34	0.6210 (5)	0.62570 (16)	0.61016 (16)	0.0859 (12)	
H34A	0.6260	0.6597	0.5842	0.129*	
H34B	0.6804	0.6320	0.6556	0.129*	
H34C	0.5006	0.6160	0.6101	0.129*	
C35	0.3141 (6)	0.38195 (18)	0.19206 (19)	0.1043 (13)	
H35A	0.2725	0.3744	0.2332	0.156*	
H35B	0.2929	0.3493	0.1631	0.156*	
H35C	0.2530	0.4143	0.1700	0.156*	
C36	0.9621 (5)	0.59350 (15)	0.29735 (18)	0.0885 (12)	
H36A	0.9487	0.5843	0.3424	0.133*	
H36B	0.9630	0.5591	0.2718	0.133*	
H36C	0.8660	0.6171	0.2767	0.133*	
H5	0.332 (3)	0.5805 (10)	0.2794 (14)	0.080*	
H2	0.322 (4)	0.6604 (9)	0.8149 (14)	0.080*	
H8	1.111 (4)	0.6578 (5)	0.3071 (16)	0.080*	

### Atomic displacement parameters (Å^2^)

**Table:**

	*U*^11^	*U*^22^	*U*^33^	*U*^12^	*U*^13^	*U*^23^
N1	0.0622 (17)	0.0529 (17)	0.0454 (17)	0.0059 (14)	0.0117 (14)	0.0018 (13)
N2	0.0665 (18)	0.0524 (17)	0.0481 (18)	0.0108 (14)	0.0130 (14)	0.0002 (14)
N3	0.0622 (18)	0.0633 (18)	0.0543 (19)	−0.0036 (15)	0.0084 (15)	−0.0047 (15)
N4	0.0623 (18)	0.0714 (19)	0.0503 (18)	−0.0068 (15)	0.0176 (15)	−0.0111 (15)
N5	0.065 (2)	0.070 (2)	0.0523 (19)	0.0044 (16)	0.0185 (15)	−0.0103 (15)
N6	0.073 (2)	0.075 (2)	0.058 (2)	−0.0117 (17)	0.0062 (16)	0.0013 (17)
O1	0.0999 (18)	0.0718 (16)	0.0644 (16)	0.0451 (14)	0.0085 (15)	0.0004 (13)
O2	0.1041 (19)	0.0646 (15)	0.0652 (16)	0.0290 (14)	0.0249 (13)	−0.0010 (12)
O3	0.0746 (16)	0.0834 (17)	0.0538 (16)	0.0110 (13)	0.0011 (12)	−0.0018 (13)
O4	0.0840 (17)	0.0817 (16)	0.0571 (14)	0.0341 (14)	0.0109 (13)	0.0050 (13)
O5	0.0789 (17)	0.0893 (18)	0.0877 (18)	0.0206 (15)	0.0171 (14)	−0.0301 (14)
O6	0.0779 (16)	0.0812 (17)	0.0595 (17)	0.0227 (13)	0.0001 (13)	−0.0020 (13)
O7	0.0803 (19)	0.111 (2)	0.106 (2)	0.0428 (17)	−0.0134 (15)	−0.0483 (17)
O8	0.0804 (18)	0.0752 (17)	0.111 (2)	0.0111 (17)	0.0290 (15)	−0.0145 (17)
C1	0.141 (4)	0.122 (3)	0.061 (3)	0.038 (3)	0.018 (2)	−0.025 (2)
C2	0.0460 (19)	0.056 (2)	0.051 (2)	0.0013 (16)	0.0081 (16)	0.0001 (17)
C3	0.054 (2)	0.052 (2)	0.058 (2)	0.0100 (16)	0.0113 (17)	−0.0076 (17)
C4	0.0509 (19)	0.0454 (19)	0.057 (2)	0.0111 (16)	0.0084 (16)	0.0018 (16)
C5	0.0436 (18)	0.0411 (18)	0.052 (2)	0.0047 (14)	0.0102 (15)	0.0003 (15)
C6	0.054 (2)	0.0452 (19)	0.064 (2)	0.0073 (16)	0.0128 (17)	−0.0025 (17)
C7	0.055 (2)	0.051 (2)	0.065 (2)	0.0115 (16)	0.0036 (18)	0.0029 (17)
C8	0.054 (2)	0.0438 (19)	0.057 (2)	0.0027 (16)	0.0184 (17)	−0.0041 (16)
C9	0.059 (2)	0.048 (2)	0.054 (2)	0.0070 (17)	0.0181 (18)	0.0089 (17)
C10	0.0499 (19)	0.0454 (19)	0.049 (2)	0.0026 (15)	0.0161 (15)	0.0003 (16)
C11	0.057 (2)	0.053 (2)	0.061 (2)	0.0087 (16)	0.0156 (17)	0.0002 (17)
C12	0.056 (2)	0.054 (2)	0.059 (2)	0.0055 (16)	0.0054 (17)	0.0075 (17)
C13	0.0464 (19)	0.0447 (19)	0.053 (2)	−0.0072 (15)	0.0131 (16)	−0.0031 (16)
C14	0.058 (2)	0.051 (2)	0.058 (2)	0.0076 (17)	0.0125 (17)	−0.0030 (17)
C15	0.057 (2)	0.057 (2)	0.056 (2)	0.0081 (17)	0.0094 (17)	0.0034 (17)
C16	0.108 (3)	0.088 (3)	0.060 (3)	0.008 (2)	0.003 (2)	0.007 (2)
C17	0.079 (3)	0.077 (2)	0.067 (2)	−0.010 (2)	0.019 (2)	−0.018 (2)
C18	0.086 (3)	0.092 (3)	0.060 (2)	0.011 (2)	0.013 (2)	−0.015 (2)
C19	0.053 (2)	0.054 (2)	0.052 (2)	0.0014 (17)	0.0064 (17)	−0.0031 (17)
C20	0.0523 (19)	0.0474 (19)	0.055 (2)	0.0057 (16)	0.0135 (16)	−0.0053 (16)
C21	0.0458 (19)	0.052 (2)	0.050 (2)	0.0017 (16)	0.0113 (16)	0.0011 (16)
C22	0.0448 (19)	0.055 (2)	0.055 (2)	0.0002 (16)	0.0167 (16)	−0.0076 (17)
C23	0.067 (2)	0.063 (2)	0.068 (3)	0.0169 (19)	0.0171 (19)	−0.0124 (19)
C24	0.065 (2)	0.067 (2)	0.072 (3)	0.0197 (19)	0.0073 (19)	−0.004 (2)
C25	0.055 (2)	0.062 (2)	0.061 (2)	−0.0026 (18)	0.0235 (18)	−0.0105 (18)
C26	0.055 (2)	0.064 (2)	0.068 (2)	−0.0046 (19)	0.0207 (19)	−0.010 (2)
C27	0.0464 (19)	0.053 (2)	0.055 (2)	−0.0017 (16)	0.0147 (16)	−0.0066 (17)
C28	0.059 (2)	0.045 (2)	0.079 (3)	−0.0008 (17)	0.0142 (19)	−0.0073 (19)
C29	0.055 (2)	0.058 (2)	0.073 (3)	0.0027 (17)	0.0046 (19)	0.0102 (19)
C30	0.0457 (19)	0.057 (2)	0.059 (2)	−0.0125 (17)	0.0114 (17)	0.0021 (19)
C31	0.060 (2)	0.055 (2)	0.062 (2)	0.0021 (17)	0.0131 (18)	−0.0107 (18)
C32	0.059 (2)	0.057 (2)	0.057 (2)	0.0045 (17)	0.0085 (17)	−0.0022 (17)
C33	0.083 (3)	0.102 (3)	0.069 (3)	−0.013 (2)	−0.001 (2)	0.021 (2)
C34	0.091 (3)	0.108 (3)	0.061 (2)	−0.011 (3)	0.018 (2)	−0.015 (2)
C35	0.104 (3)	0.127 (4)	0.080 (3)	0.014 (3)	0.014 (2)	−0.001 (3)
C36	0.090 (3)	0.080 (3)	0.089 (3)	−0.003 (2)	0.000 (2)	−0.017 (2)

### Geometric parameters (Å, º)

**Table:**

N1—C8	1.280 (3)	C12—H12	0.9300
N1—N2	1.377 (3)	C13—C14	1.397 (4)
N2—C9	1.352 (4)	C14—C15	1.374 (4)
N2—H2	0.903 (10)	C14—H14	0.9300
N3—C13	1.362 (3)	C15—H15	0.9300
N3—C16	1.444 (4)	C16—H16A	0.9600
N3—C17	1.452 (4)	C16—H16B	0.9600
N4—C25	1.287 (4)	C16—H16C	0.9600
N4—N5	1.386 (3)	C17—H17A	0.9600
N5—C26	1.352 (4)	C17—H17B	0.9600
N5—H5	0.905 (10)	C17—H17C	0.9600
N6—C30	1.371 (4)	C18—H18A	0.9600
N6—C34	1.448 (4)	C18—H18B	0.9600
N6—C33	1.451 (4)	C18—H18C	0.9600
O1—C4	1.351 (3)	C19—C20	1.370 (4)
O1—H1	0.8200	C19—C24	1.391 (4)
O2—C9	1.236 (3)	C20—C21	1.387 (4)
O3—C2	1.371 (3)	C20—H20	0.9300
O3—C1	1.419 (4)	C21—C22	1.402 (4)
O4—C21	1.353 (3)	C22—C23	1.388 (4)
O4—H4	0.8200	C22—C25	1.430 (4)
O5—C26	1.238 (3)	C23—C24	1.373 (4)
O6—C19	1.362 (3)	C23—H23	0.9300
O6—C18	1.418 (3)	C24—H24	0.9300
O7—C35	1.389 (4)	C25—H25	0.9300
O7—H7	0.8200	C26—C27	1.478 (4)
O8—C36	1.387 (4)	C27—C28	1.381 (4)
O8—H8	0.848 (10)	C27—C32	1.396 (4)
C1—H1A	0.9600	C28—C29	1.372 (4)
C1—H1B	0.9600	C28—H28	0.9300
C1—H1C	0.9600	C29—C30	1.399 (4)
C2—C3	1.370 (4)	C29—H29	0.9300
C2—C7	1.385 (4)	C30—C31	1.397 (4)
C3—C4	1.386 (4)	C31—C32	1.373 (4)
C3—H3	0.9300	C31—H31	0.9300
C4—C5	1.393 (4)	C32—H32	0.9300
C5—C6	1.392 (4)	C33—H33A	0.9600
C5—C8	1.445 (4)	C33—H33B	0.9600
C6—C7	1.366 (4)	C33—H33C	0.9600
C6—H6	0.9300	C34—H34A	0.9600
C7—H7A	0.9300	C34—H34B	0.9600
C8—H8A	0.9300	C34—H34C	0.9600
C9—C10	1.475 (4)	C35—H35A	0.9600
C10—C11	1.384 (4)	C35—H35B	0.9600
C10—C15	1.394 (4)	C35—H35C	0.9600
C11—C12	1.372 (4)	C36—H36A	0.9600
C11—H11	0.9300	C36—H36B	0.9600
C12—C13	1.398 (4)	C36—H36C	0.9600
			
C8—N1—N2	117.5 (3)	H17A—C17—H17B	109.5
C9—N2—N1	119.1 (3)	N3—C17—H17C	109.5
C9—N2—H2	123 (2)	H17A—C17—H17C	109.5
N1—N2—H2	118 (2)	H17B—C17—H17C	109.5
C13—N3—C16	121.4 (3)	O6—C18—H18A	109.5
C13—N3—C17	121.1 (3)	O6—C18—H18B	109.5
C16—N3—C17	116.4 (3)	H18A—C18—H18B	109.5
C25—N4—N5	116.1 (3)	O6—C18—H18C	109.5
C26—N5—N4	119.6 (3)	H18A—C18—H18C	109.5
C26—N5—H5	123 (2)	H18B—C18—H18C	109.5
N4—N5—H5	117 (2)	O6—C19—C20	124.5 (3)
C30—N6—C34	121.2 (3)	O6—C19—C24	114.9 (3)
C30—N6—C33	120.7 (3)	C20—C19—C24	120.5 (3)
C34—N6—C33	117.7 (3)	C19—C20—C21	119.9 (3)
C4—O1—H1	109.5	C19—C20—H20	120.1
C2—O3—C1	118.5 (3)	C21—C20—H20	120.1
C21—O4—H4	109.5	O4—C21—C20	117.0 (3)
C19—O6—C18	118.6 (2)	O4—C21—C22	121.9 (3)
C35—O7—H7	109.5	C20—C21—C22	121.1 (3)
C36—O8—H8	113 (2)	C23—C22—C21	117.0 (3)
O3—C1—H1A	109.5	C23—C22—C25	120.0 (3)
O3—C1—H1B	109.5	C21—C22—C25	122.9 (3)
H1A—C1—H1B	109.5	C24—C23—C22	122.6 (3)
O3—C1—H1C	109.5	C24—C23—H23	118.7
H1A—C1—H1C	109.5	C22—C23—H23	118.7
H1B—C1—H1C	109.5	C23—C24—C19	118.8 (3)
C3—C2—O3	124.6 (3)	C23—C24—H24	120.6
C3—C2—C7	120.3 (3)	C19—C24—H24	120.6
O3—C2—C7	115.1 (3)	N4—C25—C22	122.1 (3)
C2—C3—C4	119.7 (3)	N4—C25—H25	118.9
C2—C3—H3	120.1	C22—C25—H25	118.9
C4—C3—H3	120.1	O5—C26—N5	121.6 (3)
O1—C4—C3	117.5 (3)	O5—C26—C27	121.7 (3)
O1—C4—C5	121.3 (3)	N5—C26—C27	116.7 (3)
C3—C4—C5	121.2 (3)	C28—C27—C32	116.5 (3)
C6—C5—C4	117.2 (3)	C28—C27—C26	118.4 (3)
C6—C5—C8	120.3 (3)	C32—C27—C26	125.1 (3)
C4—C5—C8	122.4 (3)	C29—C28—C27	122.5 (3)
C7—C6—C5	122.2 (3)	C29—C28—H28	118.7
C7—C6—H6	118.9	C27—C28—H28	118.7
C5—C6—H6	118.9	C28—C29—C30	121.0 (3)
C6—C7—C2	119.3 (3)	C28—C29—H29	119.5
C6—C7—H7A	120.3	C30—C29—H29	119.5
C2—C7—H7A	120.3	N6—C30—C31	121.4 (3)
N1—C8—C5	120.4 (3)	N6—C30—C29	121.9 (3)
N1—C8—H8A	119.8	C31—C30—C29	116.7 (3)
C5—C8—H8A	119.8	C32—C31—C30	121.6 (3)
O2—C9—N2	120.3 (3)	C32—C31—H31	119.2
O2—C9—C10	122.2 (3)	C30—C31—H31	119.2
N2—C9—C10	117.5 (3)	C31—C32—C27	121.6 (3)
C11—C10—C15	116.0 (3)	C31—C32—H32	119.2
C11—C10—C9	119.4 (3)	C27—C32—H32	119.2
C15—C10—C9	124.6 (3)	N6—C33—H33A	109.5
C12—C11—C10	122.5 (3)	N6—C33—H33B	109.5
C12—C11—H11	118.8	H33A—C33—H33B	109.5
C10—C11—H11	118.8	N6—C33—H33C	109.5
C11—C12—C13	121.6 (3)	H33A—C33—H33C	109.5
C11—C12—H12	119.2	H33B—C33—H33C	109.5
C13—C12—H12	119.2	N6—C34—H34A	109.5
N3—C13—C14	121.9 (3)	N6—C34—H34B	109.5
N3—C13—C12	121.9 (3)	H34A—C34—H34B	109.5
C14—C13—C12	116.1 (3)	N6—C34—H34C	109.5
C15—C14—C13	121.6 (3)	H34A—C34—H34C	109.5
C15—C14—H14	119.2	H34B—C34—H34C	109.5
C13—C14—H14	119.2	O7—C35—H35A	109.5
C14—C15—C10	122.2 (3)	O7—C35—H35B	109.5
C14—C15—H15	118.9	H35A—C35—H35B	109.5
C10—C15—H15	118.9	O7—C35—H35C	109.5
N3—C16—H16A	109.5	H35A—C35—H35C	109.5
N3—C16—H16B	109.5	H35B—C35—H35C	109.5
H16A—C16—H16B	109.5	O8—C36—H36A	109.5
N3—C16—H16C	109.5	O8—C36—H36B	109.5
H16A—C16—H16C	109.5	H36A—C36—H36B	109.5
H16B—C16—H16C	109.5	O8—C36—H36C	109.5
N3—C17—H17A	109.5	H36A—C36—H36C	109.5
N3—C17—H17B	109.5	H36B—C36—H36C	109.5

### Hydrogen-bond geometry (Å, º)

**Table:**

*D*—H···*A*	*D*—H	H···*A*	*D*···*A*	*D*—H···*A*
O1—H1···N1	0.82	1.86	2.588 (3)	147
O4—H4···N4	0.82	1.93	2.649 (3)	146
O7—H7···O5	0.82	1.87	2.671 (3)	166
O8—H8···O2^i^	0.85 (1)	1.86 (1)	2.713 (3)	179 (4)
N5—H5···O8^ii^	0.91 (1)	2.02 (1)	2.892 (4)	161 (3)
N2—H2···O7^iii^	0.90 (1)	2.01 (2)	2.876 (3)	159 (3)

Symmetry codes: (i) *x*+1, −*y*+3/2, *z*−1/2; (ii) *x*−1, *y*, *z*; (iii) −*x*+1, −*y*+1, −*z*+1.
